# J-2156, a small molecule somatostatin type 4 receptor agonist, alleviated hindpaw hypersensitivity in the streptozotocin-induced rat model of painful diabetic neuropathy but with a 2-fold decrease in potency at an advanced stage in the model, mimicking morphine

**DOI:** 10.3389/fphar.2024.1346801

**Published:** 2024-01-22

**Authors:** A. Kuo, M. Z. Imam, R. Li, L. Lin, A. Raboczyj, A. E. Bohmer, J. R. Nicholson, L. Corradini, M. T. Smith

**Affiliations:** ^1^ School of Biomedical Sciences, Faculty of Medicine, The University of Queensland, St Lucia, QLD, Australia; ^2^ Boehringer Ingelheim Pharma GmbH & Co. KG, Biberach an der Riss, Germany

**Keywords:** SST4 receptor, J-2156, painful diabetic neuropathy (PDN), rat model, anti-allodynia, pain relief, morphine

## Abstract

There is a large unmet need for novel pain-killers to improve relief of painful diabetic neuropathy (PDN). Herein, we assessed the efficacy of the somatostatin type 4 (SST_4_) receptor agonist, J-2156, for relief of PDN in rats. Diabetes was induced with streptozotocin (STZ; 70 mg/kg) and bilateral hindpaw hypersensitivity was fully developed by 8-week post-STZ. In the intervals, 8–12-weeks (morphine-sensitive phase; Phase 1) and 16–18-weeks (morphine-hyposensitive phase; Phase 2) post-STZ, rats received a single dose of intraperitoneal (i.p.) J-2156 (10, 20, 30 mg/kg), gabapentin (100 mg/kg i.p.), subcutaneous morphine (1 mg/kg) or vehicle. Hindpaw withdrawal thresholds (PWTs) were assessed using von Frey filaments pre-dose and at regular intervals over 3-h post-dose. In Phase 1, J-2156 at 30 mg/kg evoked significant anti-allodynia in the hindpaws with maximal effect at 1.5 h compared with 1 h for gabapentin and morphine. The durations of action for all three compounds were greater than 3 h. The corresponding mean (±SEM) extent and duration of anti-allodynia (ΔPWT AUC) for gabapentin did not differ significantly from that for J-2156 (30 mg/kg) or morphine. However, in Phase 2, the ΔPWT AUC for morphine was reduced to approximately 25% of that in Phase 1, mirroring our previous work. Similarly, the mean (±SEM) ΔPWT AUC for J-2156 (30 mg/kg) in Phase 2 was approximately 45% of that for Phase 1 whereas for gabapentin the mean (±SEM) ΔPWT AUCs did not differ significantly (*p* > 0.05) between the two phases. Our findings further describe the preclinical pain relief profile of J-2156 and complement previous work in rat models of inflammatory pain, neuropathic pain and low back pain. SST_4_ receptor agonists hold promise as novel therapeutics for the relief of PDN, a type of peripheral neuropathic pain that is often intractable to relief with clinically used drug treatment options.

## 1 Introduction

Painful diabetic neuropathy (PDN) is a longterm complication of diabetes that affects 15%–25% of patients (Jang and Oh, 2023). This type of neuropathic pain is often difficult to alleviate with medications recommended by the Neuropathic Pain Special Interest Group (NeuPSIG) of the International Association for the Study of Pain (Finnerup et al., 2015). First-line medications recommended by NeuPSIG include anticonvulsants (e.g., pregabalin, gabapentin) tricyclic antidepressants (e.g., amitriptyline), serotonin-noradrenaline reuptake inhibitors (e.g., duloxetine and venlafaxine), but patients may experience dose-limiting side-effects (Finnerup et al., 2015). Hence, there is a large unmet medical need for highly efficacious novel analgesics that are well-tolerated for improving relief of PDN.

It is important to examine new targets for development of novel analgesic agents. To this end, in previous work by us and others, agonists of the somatostatin type 4 (SST_4_) receptor were shown to have promising pain relief efficacy in rodent models of inflammatory pain (Pintér et al., 2002; Sándor et al., 2006; Schuelert et al., 2015; Kántás et al., 2019), neuropathic pain (Pintér et al., 2002; Szolcsányi et al., 2004; Sándor et al., 2006; Szőke et al., 2020; Kántás et al., 2021), breast cancer induced bone pain (Shenoy et al., 2018) and low back pain (Park et al., 2019). Interestingly, in a preliminary report, single intraperitoneal (i.p.) doses of TT-232, a stable, peripherally-acting heptapeptide (D-Phe-Cys-Tyr-D-trp-Lys-Cys-Thr-NH_2_) agonist of the SST_4_ receptor, were shown to attenuate mechanical allodynia in the hindpaws of the streptozotocin-induced diabetic rat model of PDN at an early stage of this model (5-weeks post-STZ) (Szolcsányi et al., 2004).

The SST_4_ receptor, like the mu-opioid (MOP) receptor, is a member of the 7-transmembrane domain spanning superfamily of G-protein coupled receptors (Shenoy et al., 2018). This is notable, as we showed previously that the anti-allodynic efficacy of single doses of the prototypic MOP receptor agonist, morphine, that is evident up to 10–12-weeks post-STZ administration (so-called morphine-sensitive phase) is markedly reduced by 14-weeks and maintained in STZ-diabetic rats for at least 24-weeks of STZ-diabetes (so-called morphine-hyposensitive phase) in the same animals (Nielsen et al., 2007; Otto et al., 2011; Lotfipour and Smith, 2018). Hence, in the present study we assessed the anti-allodynic efficacy of the SST_4_ receptor agonist, J-2156, [(1′S,2S)-4amino-N-(1′-carbamoyl-2′-phenylethyl)-2-123 (4″-methyl-1″-naphthalenesulfonylamino)-butanamide] in both the morphine-sensitive and morphine-hyposensitive phases of the STZ-diabetic rat model of PDN to gain insight on the extent to which the anti-allodynic effects evoked by another GPCR agonist that signals via the cyclic AMP pathway (Patel et al., 1994; Bruns et al., 1995; Shenoy et al., 2018), was altered in the morphine-hyposensitive phase (Phase 2) of this model. J-2156 was selected for use herein as it has nM affinity and high potency at the SST_4_ receptor, >300-fold selectivity over SST_1_, SST_2_, SST_3_ and SST_5_ receptor types and a good off-target profile (Engström et al., 2005; Shenoy et al., 2018). J-2156 also has a low propensity to cause SST_4_ receptor desensitization (Engström et al., 2005; Engström et al., 2006) and a limited ability to cross the blood-brain-barrier (Schuelert et al., 2015).

Hence, the aims of our study described herein were to assess the time course and duration of action of single bolus doses of the selective SST_4_ receptor agonist, J-2156, in each of the morphine-sensitive (Phase 1) and morphine-hyposensitive (Phase 2) phases of the STZ-diabetic rat model of PDN and to compare these outcomes with that of the positive control, gabapentin, the reference control, morphine and the negative control, vehicle.

## 2 Materials and methods

### 2.1 Drugs, chemicals, and reagents

Streptozotocin (STZ), citric acid and trisodium citrate were from Sigma Aldrich (Sydney, Australia). Morphine sulphate injections were from Hospira (Brisbane, Australia). Gabapentin was provided by Dr Ben Ross (School of Pharmacy, The University of Queensland). Sodium benzylpenicillin (Benpen™) vials containing 3 g of powder, were from CSL Limited (Sydney, Australia). Sterile water for injection (BP) vials were from Pfizer (Sydney, Australia). J-2156 was supplied by Boehringer Ingelheim Pharma GmbH & Co. KG, Biberach an der Riss, Germany. Single lumen polyethylene tubing (0.5-mm internal diameter) was from SDR Scientific Pty Ltd. (Chatswood, Australia). Accu-Chek^®^ Guide glucometer and blood glucose testing strips (Accu-Chek^®^ Guide) were from Roche Diabetes Care Australia Pty. Ltd. (Bella Vista, Australia). Isoflurane (Isothesia^®^ NXT) was from Provet AU (Northgate, Australia). Medical grade CO_2_ and O_2_ were from Coregas Australia Ltd. (Brisbane, Australia).

### 2.2 Animals

Ethics approval was given by the Animal Ethics Committee of The University of Queensland (Brisbane, Australia) (Approval No. SBS/303/20). This study was conducted in accordance with the guidelines set out in the Australian Code of Practice for the Care and Use of Animals for Scientific Purposes (8^th^ edition, 2013). Male Wistar rats were from the Animal Resources Centre (Perth, Australia). Rats were housed in groups of 2–3 in a temperature-controlled room (21 ± 2°C; mean ± SD) with a 12 h/12 h light/dark cycle. Environmental enrichment comprised chewsticks, Kim-wipes and rodent hutches constructed from red perspex. Rats had *ad libitum* access to rodent chow and water, and were acclimatized for several days prior to initiation of experimentation.

### 2.3 Induction of diabetes with streptozotocin

Rats were anesthetized with 3% isoflurane delivered in oxygen by inhalation to enable insertion of a polyethylene cannula (pre-filled with 0.2 mL of heparinized sterile saline) into the right common jugular vein (Wei et al., 2003). A small amount of blood was withdrawn to verify that the cannula was correctly placed in the vein. Diabetes was induced by injection of a freshly made STZ solution (70 mg/kg in 20 mM citrate buffer at pH 4.5) via the jugular vein cannula. Benzylpenicillin (60 mg s.c.) was administered and rats were kept warm and monitored during surgical recovery. Diabetes was confirmed on day 9 post-STZ in individual rats if blood glucose levels (BGLs) were ≥15 mM.

### 2.4 Assessment of body weight as an index of general health

Body weights were measured at regular intervals in individual rats during the 18-week post-STZ study duration, as an index of general health.

### 2.5 Pain behavioral testing

#### 2.5.1 Assessment of paw withdrawal thresholds in the bilateral hindpaws

Paw withdrawal thresholds (PWTs) in the bilateral hindpaws were assessed using calibrated Von Frey filaments (2–20 g) (Stoelting Co., Wood Dale, IL, United States) using the up-down method employed routinely in our laboratory (Shenoy et al., 2018). In brief, rats were placed individually into wire mesh cages (20 cm × 20 cm × 20 cm) and allowed to acclimatise. Von Frey filaments were used to measure the lowest mechanical threshold to evoke a brisk hindpaw withdrawal reflex starting with the 6 g filament and then selecting filaments in 2 g increments up or down depending upon the response. The absence of a response after approximately 3 s prompted application of the next filament of increasing force. A score of 20 g was given to animals that did not respond to any of the filaments. Baseline PWTs were measured prior to the induction of diabetes with STZ and then at 1, 2, 4, 6, 8, 10, 12, 14, 16, and 18 weeks post-STZ.

### 2.6 Administration of J-2156, morphine or vehicle and pain behavioral testing

In each of the intervals, 8–12-weeks (morphine-sensitive phase; Phase 1) and 16–18-weeks (morphine-hyposensitive phase; Phase 2) post-STZ administration, rats were assigned randomly to receive a single dose of subcutaneous morphine (1 mg/kg, s.c.), J-2156 (10, 20, 30 mg/kg, i.p.) or gabapentin (100 mg/kg i.p.) or vehicle (sterile water for injection; 1.0 mL/kg i.p.). The doses of J-2156 utilized spanned the efficacious dose range for the relief of mechanical allodynia and mechanical hyperalgesia in a rat model of breast cancer induced bone pain (Shenoy et al., 2018) and mechanical hyperalgesia in a rat model of chronic low back pain (Park et al., 2019).

Dosing was according to a “washout protocol” such that individual rats received up to 5 single doses of one test item in each of Phases 1 and 2 of the study, with at least a 3-day washout between successive doses. Von Frey filaments were used to measure pre-dosing paw withdrawal thresholds (PWTs) in the bilateral hindpaws, and were the mean of three readings taken approximately 5 min apart for each hindpaw. For each test item dose administered, bilateral hindpaw von Frey PWTs were measured pre-dose and at the following times post-dosing times: 0.25, 0.5, 0.75, 1.0, 1.5, 2.0, and 3.0 , by testers blinded to treatment group.

### 2.7 Data and statistical analysis

Mean (±SEM) PWT *versus* time curves were generated. The post-dose change in PWT (ΔPWT) values for individual rats were calculated by subtracting the mean (±SEM) pre-dosing baseline PWT value from each post-dose value and ΔPWT *versus* time curves were generated. Any negative ΔPWT values were arbitrarily assigned a value of 0. Mean (±SEM) ΔPWT *versus* time curves were plotted for each dose of each test item and the extent and duration of anti-allodynia (area under the ΔPWT *versus* time curve; ΔPWT AUC) values were estimated for individual rats using trapezoidal integration. The Mann-Whitney test was used to compare ΔPWT AUC values between Phases 1 and 2 of the STZ-diabetic rat model of PDN, for each of the test items assessed. The GraphPad™ Prism statistical analysis software package (v 9.0.0) was used to generate graphs and for data and statistical analysis. The statistical significance criterion was *p* ≤ 0.05.

## 3 Results

### 3.1 Induction of STZ-diabetes

**FIGURE 1:**
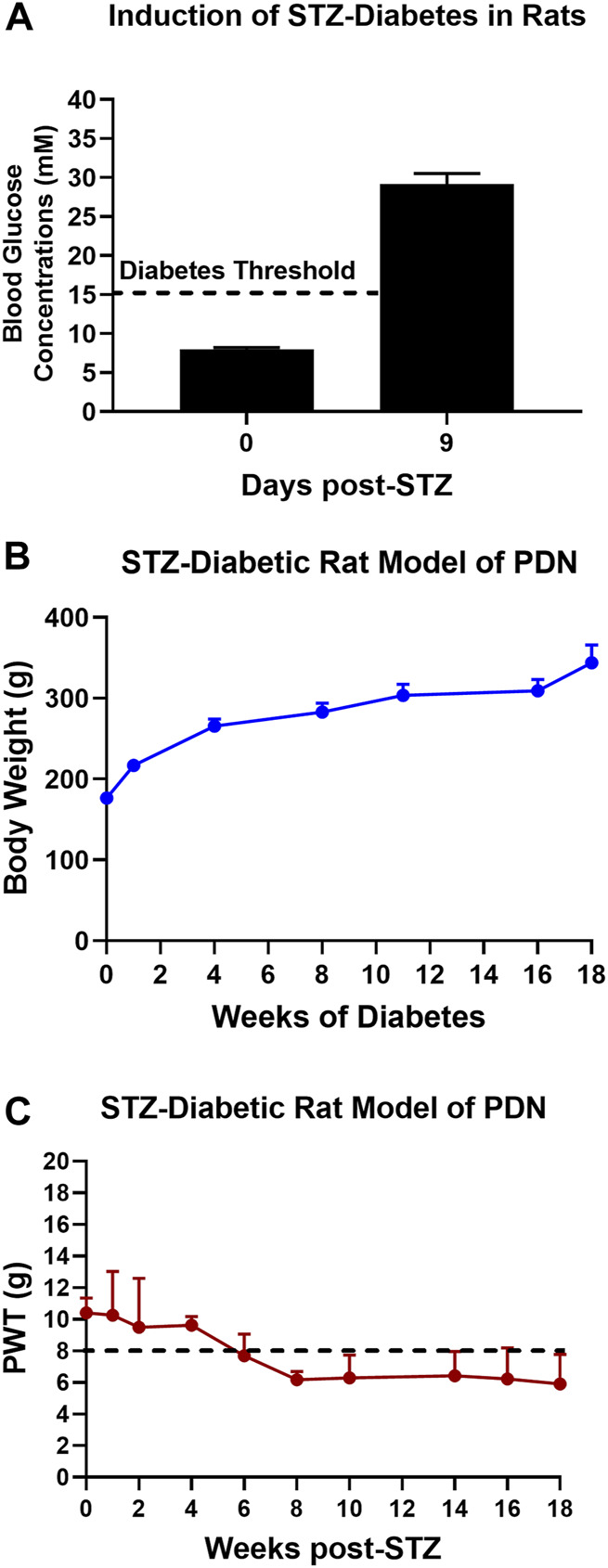
**(A)** Blood glucose concentrations on day 9 after STZ-diabetes induction in rats were >15 mM confirming that rats were diabetic. **(B)** There was a temporal increase in mean (±SEM) body weights of STZ-diabetic rats for the 18-weeks post-STZ study duration, indicative of good general health. **(C)** Temporal development of mechanical hypersensitivity in the bilateral hindpaws with mechanical allodynia fully developed (PWTs ≤ 8 g) by 8-weeks post-STZ which was maintained until study completion at 18-weeks post-STZ.

The mean (±SEM) blood glucose concentration was 8.0 (±0.3) mM just prior to the induction of diabetes and by day 9 post-STZ, the mean (±SEM) blood glucose concentration was 29.1 (±1.4) mM, confirming the development of marked diabetes in these animals (Figure 1A).

### 3.2 General health

The mean (±SEM) body weight of rats was 176.4 (±2.1) g just prior to the induction of diabetes with STZ. Thereafter, there was a temporal increased in mean (±SEM) body weight during the study period (Figure 1B) attesting to the satisfactory general health of these animals until study completion.

### 3.3 Temporal development of mechanical hypersensitivity in the bilateral hindpaws

After the induction of diabetes with STZ, there was progressive development of mechanical hypersensitivity in the bilateral hindpaws such that mechanical allodynia was fully developed (PWTs ≤ 8 g) by 8-weeks post-STZ which was maintained until study completion at 18-weeks post-STZ (Figure 1C).

### 3.4 Anti-allodynic effects of morphine in STZ-diabetic rats

**FIGURE 2:**
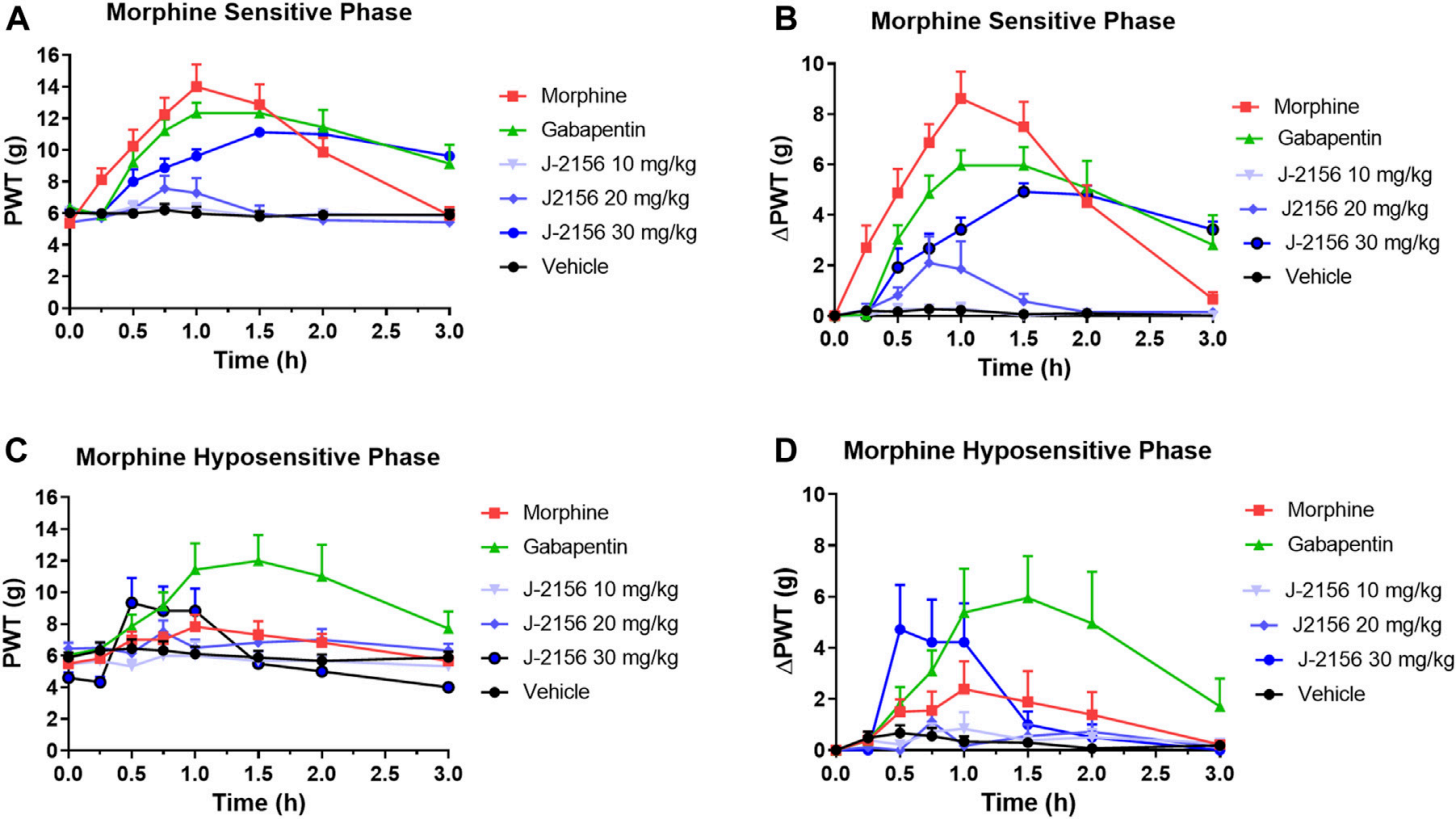
**(A)** Mean (±SEM) paw withdrawal threshold (PWT) *versus* time curves for J-2156 at 10, 20, and 30 mg/kg i.p. (*n* = 7–10/group), morphine at 1 mg/kg s.c. (*n* = 8), gabapentin at 100 mg/kg i.p. (*n* = 9) or vehicle (1 mL/kg i.p., *n* = 10) in the morphine-sensitive phase (8–12-weeks post-STZ; Phase 1). **(B)** Mean (±SEM) change in paw withdrawal threshold (ΔPWT) *versus* time curves for J-2156 at 10, 20, and 30 mg/kg i.p. (*n* = 7–10/group), morphine at 1 mg/kg s.c. (*n* = 8), gabapentin at 100 mg/kg i.p. (*n* = 9) or vehicle (1 mL/kg i.p., *n* = 10) in Phase 1. **(C)** Mean (±SEM) paw withdrawal threshold (PWT) *versus* time curves for J-2156 at 10, 20 and 30 mg/kg i.p. (*n* = 6/group), morphine at 1 mg/kg s.c. (*n* = 6), gabapentin at 100 mg/kg i.p. (*n* = 7) or vehicle (1 mL/kg i.p., *n* = 9) in the morphine-hyposensitive phase (16–18 weeks post-STZ; Phase 2). **(D)** Mean (±SEM) change in paw withdrawal threshold (ΔPWT) *versus* time curves for J-2156 at 10, 20, and 30 mg/kg i.p. (*n* = 6/group), morphine at 1 mg/kg s.c. (*n* = 6), gabapentin at 100 mg/kg i.p. (*n* = 7) or vehicle (1 mL/kg i.p., *n* = 9) in Phase 2.

**FIGURE 3:**
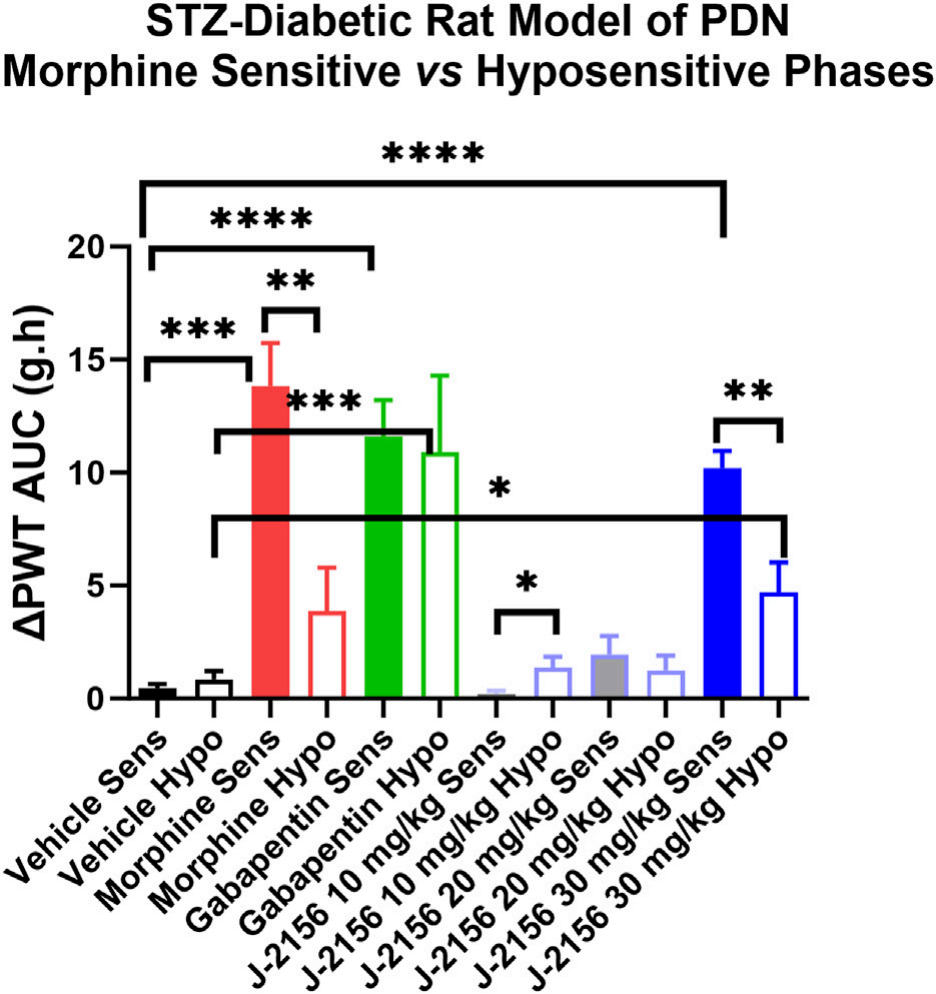
Mean extent and duration of anti-allodynia (ΔPWT AUC values) evoked by single bolus doses of J-2156 (10, 20, 30 mg/kg i.p.), morphine (1 mg/kg s.c.), gabapentin (100 mg/kg i.p.) and vehicle (1 mL/kg i.p.) in Phase 1 (8–12 weeks post-STZ) and Phase 2 (16–18 weeks post-STZ) of the STZ-diabetic rat model of PDN. The mean (±SEM) ΔPWT AUC values for J-2156 dose at 30 mg/kg i.p. and for gabapentin (100 mg/kg i.p.) were significantly different from vehicle for both the morphine sensitive and morphine hyposensitive phases whereas the mean (±SEM) ΔPWT AUC for morphine in the hyposensitive phase was not significantly different from the corresponding value for vehicle. *<0.05, **<0.01, ***<0.001, ***<0.0001.

Single doses of morphine (1 mg/kg) administered to STZ-diabetic rats during Phase 1, evoked anti-allodynia characterized by a rapid onset of action with the mean (±SEM) peak effect at 1 h post-dose with a mean duration of action of 3 h (Figures 2A,B). The mean (±SEM) extent and duration of anti-allodynia (ΔPWT AUC) was 13.8 (±1.9) g.h which differed significantly (*p* < 0.05) from that for vehicle (0.8 ± 0.2 g h) (Figure 3). These data demonstrate that STZ-diabetic rats are sensitive to morphine during Phase 1 after the induction of diabetes with STZ. After administration of the same dose of morphine to STZ-diabetic rats in Phase 2, mean (±SEM) peak anti-allodynia was also at 1 h post-dose and the mean duration of action remained at 3 h (Figures 2C,D). However, there was a significant (*p* < 0.05) 3.6-fold reduction in the ΔPWT AUC value to 3.9 (±1.9) g h (Figure 3) which did not differ significantly (*p* > 0.05) from that for vehicle (0.8 ± 0.4 g h). These data clearly show temporal development of morphine hyposensitivity in STZ-diabetic rats herein mirroring previous work from our laboratory in this regard (Nielsen et al., 2007; Otto et al., 2011; Lotfipour and Smith, 2018).

### 3.5 Anti-allodynic effects of J-2156 in STZ-diabetic rats

#### 3.5.1 Phase 1

In Phase 1 of STZ-diabetes in rats herein, single i.p. doses of the SST_4_ receptor agonist, J-2156 (10–30 mg/kg), evoked dose-dependent anti-allodynia with the mean peak effect observed at 0.75 h and 1.5 h for the 20 mg/kg and 30 mg/kg doses respectively (Figures 2A,B). The corresponding durations of action were 1.5 h and >3 h respectively. The mean (±SEM) extent and duration of anti-allodynia (ΔPWT AUC) evoked by J-2156 at 30 mg/kg at 10.2 (±0.8) g.h (Figure 3) did not differ significantly (*p* > 0.05) from that determined for morphine at 1 mg/kg (13.8 ± 1.9 g h) in Phase 1 and it was significantly different from that for vehicle in Phase 1 (0.5 ± 0.2 g h) (Figure 3). By comparison, the 10 mg/kg and 20 mg/kg doses of J-2156 did not evoke significant anti-allodynia in a manner similar to vehicle. Specifically, the mean (±SEM) ΔPWT AUC values for J-2156 at 10 mg/kg and 20 mg/kg (0.2 ± 0.2 g h and 1.9 ± 1.8 g h) did not differ significantly (*p* > 0.05) from that for vehicle (0.5 ± 0.2 g h) (Figure 3). The reason for the lower potency of J-2156 in Phase 1 herein compared to that previously reported in rat models of inflammatory pain (Pintér et al., 2002; Sándor et al., 2006; Schuelert et al., 2015; Kántás et al., 2019), neuropathic pain (Pintér et al., 2002; Szolcsányi et al., 2004; Sándor et al., 2006; Szőke et al., 2020; Kántás et al., 2021), breast cancer induced bone pain (Shenoy et al., 2018) and low back pain (Park et al., 2019), may be underpinned by diabetes-induced alterations in J-2156 pharmacokinetics, but this remains to be investigated in future work. Importantly, there were no overt signs of sedation at any of the doses of J-2156 administered and CNS side-effects would not be expected as J-2156 has a limited ability to cross the blood-brain-barrier (BBB) (Schuelert et al., 2015). Nevertheless, in future work beyond the scope of this investigation, we intend to formally assess the effects of J-2156 relative to gabapentin and morphine on motor incoordination as quantitative measures of sedation using the rotarod test as described by others (Ahmad et al., 2021).

#### 3.5.2 Phase 2

For STZ-diabetic rats in Phase 2, the mean peak anti-allodynic effect of J-2156 at 30 mg/kg occurred earlier at 0.5 h and had a shorter duration of action (1.5 h) compared with the corresponding data for this dose of J-2156 administered in Phase 1 (Figures 2A,B). Although the mean (±SEM) extent and duration of anti-allodynia (ΔPWT AUC) evoked by J-2156 at 30 mg/kg at 4.7 (±1.3) g.h was approximately 2-fold lower (*p* < 0.05) than that evoked in Phase 1 (10.2 ± 0.8 g h), it was significantly different (*p* < 0.05) from the corresponding effect evoked by vehicle in Phase 2 (0.8 ± 0.4 g h) (Figure 3). The mean ΔPWT AUC values evoked by J-2156 at the 10 mg/kg and 20 mg/kg doses (1.4 ± 0.5 and 1.2 ± 0.7 g h respectively) did not differ significantly (*p* > 0.05) from that for vehicle (0.8 ± 0.4 g h) (Figure 3).

### 3.6 Anti-allodynic effects of gabapentin in STZ-diabetic rats

#### 3.6.1 Phase 1

Following administration of a single dose of the positive control, gabapentin (100 mg/kg), to STZ-diabetic rats in Phase 1 of the model, mean (±SEM) peak anti-allodynia was observed at 1 h post-dose and the duration of action was > 3 h (Figures 2A,B). The corresponding mean (±SEM) extent and duration of gabapentin anti-allodynia (ΔPWT AUC) at 11.6 (±1.6) g.h (Figure 3) did not differ significantly (*p* > 0.05) from that evoked by morphine at 1 mg/kg (13.8 ± 1.9 g h) or J-2156 at 30 mg/kg (10.2 (±0.8) g.h) in Phase 1 (Figure 3) but it was significantly different (*p* < 0.05 from that evoked by vehicle (0.8 ± 0.4 g h).

#### 3.6.2 Phase 2

For STZ-diabetic rats in Phase 2 of the model, the mean (±SEM) peak anti-allodynic effect of a single dose of gabapentin (100 mg/kg) was at 1 h post-dose and the duration of action was > 3 h (Figures 2A,B). The mean (±SEM) extent and duration of anti-allodynia (ΔPWT AUC) at 10.9 (±3.4) g.h (Figure 3) did not differ significantly (*p* > 0.05) from that determined for the same dose of gabapentin in Phase 1 (11.6 (±1.6 g h) and it was significantly different (*p* < 0.001) from that evoked by vehicle (Figure 3). Thus, our findings show that the potency of gabapentin was similar between both phases of the STZ-diabetic rat model of PDN.

## 4 Discussion

Our main finding herein is that single i.p. bolus doses (10–30 mg/kg) of the SST_4_ receptor agonist, J-2156, evoked dose-dependent relief of mechanical allodynia in the bilateral hindpaws of the STZ-diabetic rat model of PDN in the interval 8–12 weeks post-STZ (Phase 1), a period when these animals were sensitive to morphine. However, it was only at the highest dose of J-2156 tested (30 mg/kg) that the extent and duration of anti-allodynia differed significantly from that for vehicle. The anti-allodynic effects of J-2156 in Phase 1 of the STZ-diabetic rat model are aligned with a previous preliminary report by others whereby single i.p. doses (10–100 μg/kg) of the peptidic SST_4_ receptor agonist, TT-232, evoked anti-allodynia, although the time to peak effect, duration of action and the extent and duration of anti-allodynia were not reported (Szolcsányi et al., 2004).

In Phase 1 of the STZ-diabetic rat model herein, the time to peak effect and the duration of action of a single dose of J-2156 (30 mg/kg) were much shorter at 0.5 h and 1.5 h respectively relative to 1.5 h and >3 h respectively observed in the same animals in Phase 2 of the model. By comparison, the time to peak effect and the duration of action (1 h and 3 h respectively) evoked by morphine at 1 mg/kg were the same in both phases of the model. Regarding the extent and duration of anti-allodynia evoked by J-2156 (30 mg/kg) in Phase 2, it was approximately 2-fold lower than that evoked by the same dose of J-2156 in Phase 1 of the model. However, this 2-fold decrease was less than the corresponding 3.6-fold decrease in the extent and duration of anti-allodynia evoked by morphine (1 mg/kg s.c.) in Phase 2 relative to Phase 1 of this model.

It is notable that even in Phase 1 of the STZ-diabetic rat model herein, the potency of J-2156 for the relief of mechanical allodynia in the bilateral hindpaws was lower than that anticipated based upon our previous work in a rat model of breast cancer-induced bone pain (BCIBP) (Shenoy et al., 2018) and in a rat model of chronic low back pain (Park et al., 2019). In our rat model of BCIBP, the ED_50_ values for J-2156 for the relief of mechanical allodynia and mechanical hyperalgesia in the ipsilateral hindpaws were 3.7 and 8.0 mg/kg i.p. respectively (Shenoy et al., 2018). The corresponding ED_50_ values for the contralateral hindpaws were 6.6 and 5.0 mg/kg i.p. respectively (Shenoy et al., 2018). Additionally, in our rat model of chronic low back pain, the ED_50_ values for J-2156 for the relief of primary and secondary hyperalgesia in the lumbar axial deep tissues at L1 and L4/L5 were 22.7 and 18.5 mg/kg i.p. respectively (Park et al., 2019).

The 5 to 10-fold higher potency of J-2156 for the relief of mechanical allodynia and mechanical hyperalgesia in the hindpaws in a rat model of BCIBP may be due to the fact that both inflammatory and neuropathic mechanisms contribute to the pathobiology of this model (Shenoy et al., 2018). This is particularly so as the efficacious doses of J-2156 in the Complete Freund’s adjuvant and the carrageenan-induced rodent models of inflammatory pain, were much lower in the range 0.01–1.0 mg/kg (Helyes et al., 2006; Sándor et al., 2006; Schuelert et al., 2015) than the anti-allodynic dose of J-2156 (30 mg/kg) in the STZ-rat model of PDN, a peripheral neuropathic pain condition that is known to often be intractable to treatment.

In agreement with our previous longterm longitudinal experiments in the STZ-diabetic rat model of PDN (Nielsen et al., 2007; Otto et al., 2011; Lotfipour and Smith, 2018), there was temporal development of morphine hyposensitivity for relief of mechanical allodynia in the bilateral hindpaws of STZ-diabetic rats herein. Possible explanations for the 3.6-fold decrease in the anti-allodynic potency of morphine in Phase 2 of this model, include impaired basal G-protein activity in the spinal cord manifesting as an apparent reduction in agonist-stimulated MOP receptor function compared with the prediabetic state (Otto et al., 2011). As the SST_4_ receptor is also G-protein coupled, it is plausible that impaired basal G-protein activity may contribute to the approximately 2-fold reduction in the anti-allodynic potency of J-2156 observed in Phase 2 of this rat model of PDN herein.

Although morphological, biochemical and biophysical data have shown functional heterodimerization between the SST_4_ and the δ-opioid (DOP) receptor with these heterodimers modulating signaling pathways associated with pain and opioid withdrawal in a manner different from that of the parent receptors (Somvanshi and Kumar, 2014), data on co-localization and/or heterodimerization of the SST_4_ and the MOP receptor are lacking. Hence, there is a need for future work aimed at addressing these knowledge gaps along with assessment of possible differential changes in the expression and function of co-localized and/or heterodimerized SST4 and MOP receptors to modulate pro-nociceptive signaling at multiple levels of the somatosensory nervous system in Phases 1 and 2 of the STZ-diabetic rat model.

Another factor potentially contributing to the 3.6-fold and 2.0-fold decrease in potency of morphine and J-2156 in Phase 2 compared with Phase 1 of the STZ-diabetic rat model of PDN herein, is altered pharmacokinetics between the two phases. In a study of the pharmacokinetics of morphine in the STZ-diabetic rat compared with control non-diabetic rats, the plasma morphine concentrations were lower in STZ-diabetic rats at 4-weeks post-STZ administration such that the mean systemic exposure to morphine in the STZ-diabetic rats was only 58% of that in control non-diabetic rats (Hasegawa et al., 2010). However, whether there are further changes in the pharmacokinetics and metabolism of morphine in Phase 2 compared with Phase 1 of the STZ-diabetic rat, remains to be assessed.

Interestingly, the anti-allodynic potency of gabapentin did not differ between the two phases of the STZ-diabetic rat model. A factor potentially contributing to these findings is that the anti-allodynic effects of gabapentin are mediated by a non-GPCR mechanism in contrast to morphine and J-2156. Also, systemic exposure to gabapentin after administration of a single dose at 50 mg/kg i.p. at 19-days after STZ-diabetes induction in rats, did not differ significantly from that for similar STZ-diabetic rats rendered euglycemic by insulin treatment (Benzi et al., 2018). Future work is required to assess whether or not the pharmacokinetics of gabapentin remain unchanged between Phases 1 and 2 of the STZ-diabetic rat model of PDN.

In summary, single bolus doses of J-2156 at the highest dose tested (30 mg/kg i.p.) alleviated hindpaw hypersensitivity with an effect similar to that of gabapentin at 100 mg/kg i.p. and morphine at 1 mg/kg s.c. in Phase 1 of the STZ-diabetic rat model of PDN. The mechanism(s) underpinning the ∼2-fold decrease in the anti-allodynic potency of J-2156 in Phase 2 of the STZ-diabetic rat model are currently unclear. In future work beyond the scope of that described herein, we will use immunohistochemical and molecular biological methods in a range of neural tissues to gain novel insight on the contributing factors. We will also assess the extent to which there is impaired basal G-protein activity in the spinal cord to reduce agonist-stimulated SST_4_ receptor function as we have shown previously that this is a factor contributing to the development of morphine-hyposensitivity in Phase 2 of a diabetic rat model (Otto et al., 2011). Based on these findings and the fact that J-2156 is peripherally-selective with a non-opioid mechanism of action, SST_4_ receptor agonists hold promise for development as novel pain therapeutics for the relief of PDN, a type of peripheral neuropathic pain that is often intractable to relief with clinically used drug treatment options. Our findings further describe the preclinical pain relief profile of J-2156 and complement previous work in rat models of inflammatory pain, neuropathic pain, combined inflammatory and neuropathic pain (BCIBP), and chronic low back pain.

## Data Availability

The raw data supporting the conclusion of this article will be made available by the authors, without undue reservation.
